# Effect of incorporation of calcium lactate on physico-chemical, textural, and sensory properties of restructured buffalo meat loaves

**DOI:** 10.14202/vetworld.2016.151-159

**Published:** 2016-02-13

**Authors:** A. Irshad, B. D. Sharma, S. R. Ahmed, S. Talukder, O. P. Malav, Ashish Kumar

**Affiliations:** 1 Department of Livestock Products Technology, College of Veterinary and Animal Sciences, Kerala Veterinary and Animal Sciences University, Mannuthy, Thrissur - 680 651, Kerala, India; 2 Division of Livestock Products Technology, Indian Veterinary Research Institute, Izatnagar - 243 122, Uttar Pradesh, India; 3 Department of Livestock Products Technology, College of Veterinary Science, Guru Angad Dev Veterinary and Animal Sciences University, Ludhiana, Punjab, India

**Keywords:** buffalo meat, calcium fortification, Lovibond^®^ tintometer color units, meat loaves, restructured products, texture profile analysis and sensory attributes

## Abstract

**Aim:**

The present study was conducted to develop a functional meat product by fortifying calcium (in the form of calcium lactate) with restructured buffalo meat loaf (RBML).

**Materials and Methods:**

Deboned buffalo meat obtained from the carcass of adult female buffalo within 5-6 h of slaughter and stored under frozen condition. Calcium fortified RBML were prepared by replacing the lean buffalo meat with calcium lactate powder at 0%, 1%, 1.25%, and 1.5% level through the pre-standardized procedure. The developed products were evaluated for physico-chemical properties, proximate composition, calcium concentration (mg/100 g), water activity (a_w_), Lovibond^®^ tintometer color units, texture profile analysis (TPA), and sensory qualities as per-standard procedures.

**Results:**

Of the various product quality parameters evaluated, cooking yield (%), product pH, moisture (%), protein (%), fat (%), and water activity (a_w_) decreases significantly with increasing level of calcium lactate. Calcium content of fortified functional RBMLs was 135.02, 165.73, and 203.85 mg/100 g as compared to 6.48 mg/100 g in control. Most of the sensory scores at 1% and 1.25% levels of calcium lactate in treatment products remained comparable among themselves and control product, with a gradual decline.

**Conclusions:**

The present study concluded that 1.25% calcium lactate was the optimum level for the fortification of calcium in RBML without affecting the textural and sensory properties which could meet out 15% of recommended dietary allowance for calcium.

## Introduction

Interest in the dietary calcium has intensified in the recent years as a result of increased awareness about the importance of higher calcium intake [1]. Calcium is one of the most important nutrients in the human diet. In addition to conferring structural integrity to mineralized tissue (where about 99% of total calcium is found), it plays a diverse role in maintaining cellular function such as cellular metabolism, blood clotting, enzyme activation, and so on [2,3]. Meat as such is relatively poor in calcium, containing only about 10 mg/100 g of meat [4]. So, there is a dire need to fortify meat products with calcium so that a sufficient amount of recommended dietary allowance (RDA) for calcium can be met through meat products also [5]. The normal RDA for calcium in the age group of 19-50 is l000 mg/day [6,7].

The clinical implications of calcium deficiency include rickets, poor bone mass accrual as well as abnormal fetal programing during pregnancy, poor peak bone mass due to poor accrual in childhood and adolescence, postmenopausal osteoporosis, and osteoporosis of the elderly [1]. While the etiologies of all of these diseases are multi-factorial and poorly understood, there is some evidence to support the hypothesis that increased calcium intake will reduce the risk of each of the diseases [7-10]. A growing concern regarding bone health in people of all ages has prompted the food industry to respond by adding calcium to foods and beverages [11]. Various calcium containing beverages include fortified milk products, hot and cold drink mixes, orange juice, carbonated soft drinks, beer, and even water are available in the market [12]. So, there is a need to enrich meat products with calcium so that a sufficient amount of RDA for calcium can be met through meat products also [11]. Regarding meats, some work has been carried out on calcium enrichment in ground beef patties [13]; reduced fat beef emulsion [14]; pork sausage [15]; cooked meat sausage [2], and calcium enriched chicken meat rolls [11]. The ideal calcium source used to enrich foods should be highly absorbable, inexpensive, safe, and compatible with the food delivery vehicle [16]. Several salts of calcium are available, e.g. inorganic salts such as calcium carbonate, calcium chloride, calcium phosphate, and organic salts such as calcium citrate, calcium lactate, and calcium gluconate. In general, organic salts of calcium are more bioavailable than inorganic salts [17]. Many of the researchers recommended calcium lactate for the enrichment of the meat products due to its nutritional value with high calcium content (13%), bland taste, and neutral aroma [15,18-20].

India is endowed with the largest buffalo population in the world which is about 58% of the world's buffalo population. About 10.66 million buffaloes are slaughtered annually producing 1.53 million MT of buffalo meat which accounts 31% of total meat production of the country [21]. Buffalo meat has been the major one in Indian meat export accounting more than 85% of total meat export mostly in frozen form [22]. Buffalo meat is abundantly available in India and has enormous potential for development into valuable and highly palatable processed meat products. However, the production of processed buffalo meat is minimal at present. As per Agricultural and Processed Food Products Export Development Authority [23], only 2% of the total meat is processed in India. So, processing of buffalo meat is essential to exploit its undermined potential. The majority of buffaloes in India are slaughtered from aged/spent animals (about 10-15 years) after completion of their productive period resulting in tough meat with poor quality characteristics such as tough texture, less juiciness, and comparatively dark color [24,25]. This coarse textured meat needs to be subjected to special processing and cooking methods to improve tenderness [26]. Restructuring of meat enables the use of less valuable meat components to produce high-quality meat products at a reduced cost [27]. Therefore, the enhanced use of less demanded cuts and/or raw material from older maturity classes can be achieved using restructured meat technology. Meat processors and consumers can benefit from the development of efficient and economical technologies for processing buffalo meat into value-added convenience meat products with high acceptability at a reasonable cost.

Hence, the present study was carried out to develop a calcium fortified restructured buffalo meat loaves (RBMLs) by incorporating a suitable level of calcium lactate in spent buffalo meat readily available in the market.

## Materials and Methods

### Ethical approval

Since the study was conducted on the buffalo meat purchased from the local slaughter house, ethical approval from Animal Ethics Committee of the institute was not necessary.

### Location

The study was undertaken at Indian Veterinary Research Institute (IVRI), Izatnagar, Bareilly, Uttar Pradesh located at 28°10’ N, 78°23’ E, and lies in the northern region of India. The place has a humid subtropical climate with an elevation of 268 m (879 ft) above mean sea level.

### Buffalo meat and other ingredients

Deboned buffalo meat obtained from the carcass of adult female buffalo (>10 years of age) was procured from the local market of Bareilly within 5-6 h of slaughter. All visible fascia and external fat were trimmed off, and meat portions were made into cuts of approximately 0.5 kg. The cuts were then packaged separately in low-density polyethylene (LDPE) pouches and kept in the refrigerator (4±1°C) for conditioning for about 24 h. Thereafter, the samples were shifted to the deep freezer (Blue Star, FS345, Denmark) for storage at −18±2°C until further use.

To prepare condiment mix, onion, and garlic were peeled off, cut into small pieces and homogenized separately in a kitchen mixer to obtain a fine paste. For the preparation of RBMLs, onion and garlic were used in the ratio 2:1. The spice ingredients were purchased from local market, free from extraneous matter and dried in hot air oven at 50±2°C for 4 h. The ingredients were ground and sieved through a fine mesh. The powders were mixed in suitable proportion to obtain spice mixture. The spice mix was stored in a plastic container for subsequent use (Table-1). All the chemicals (analytical grade) were obtained from standard firms (Qualigen^®^, Hi-Media^®^, Sdefine^®^, etc.). Generally Recognized as Safe/Food Grade Chemicals were used for fortification were supplied by Qualigen^®^ Fine Chemicals (A division of Glaxo India Limited), Mumbai. LDPE films (200 gauges) were procured from M/s Hitkari Industries Ltd., New Delhi - 14.

**Table-1:** Composition of spice mix for RBMLs.

Ingredients	Percentage (w/w)
Coriander powder (*Dhania*)	17
Cumin seed (*Jeera*)	10
Aniseed (*Soanf*)	10
Black pepper (*Kalimirch*)	10
Caraway seed (*Ajowan*)	10
Turmeric (*Haldi*)	10
Dried ginger (*Saundh*)	10
Capsicum (*Mirch powder*)	8
Cardamom (*Badi elaichi*)	5
Cinnamon (*Dal chini*)	5
Cloves (*Laung*)	3
Nutmeg (*Jaibhal*)	1
Lace (*Jaipatri*)	1
Total	100

RBMLs=Restructured buffalo meat loaves

### Experimental design

**Figure-1:**
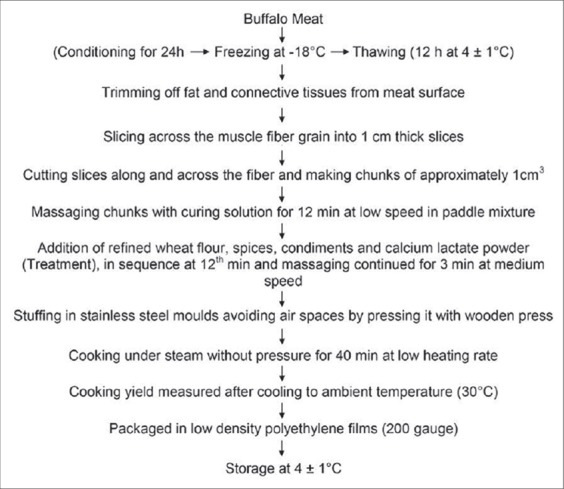
Flow chart of processing protocol for the development of restructured buffalo meat loaves.

Frozen meat was thawed (approximately 12 h at 4±1°C, reaching between −3 and −5°C). The partially thawed meat was carefully trimmed off adhering visible loose connective tissue and fascia was sliced across the grain into 1 cm thick slices. The sliced buffalo meat was then cut along and across to chunks of nearly 1 cm^3^. Temperature of the meat chunks was maintained below 2°C by keeping it immediately in a refrigerator at 0°C after chunking, so as to ensure temperature of meat chunks below 10°C throughout the processing. Meat chunks (77% of formulation) in semi-frozen state were placed in paddle mixture (HOBART, Model: N50G) and massaging was done initially at low speed with simultaneous addition of curing solution (15%) (Table-2) which facilitated the extraction of muscle proteins from meat and formed tacky exudates to bind meat pieces. After the initial 8 min of mixing at low speed, the refined wheat flour (3%), spices (2%), condiments (3%), and calcium lactate powder were added in order and concurrently mixed/blended for additional 4 min at medium speed for uniform mixing (Figure-1).

**Table-2:** Formulation of curing solution for RBMLs.

Ingredients	Quantity (g)
Sodium chloride (2%)	13.34
Cane sugar (0.76%)	5.07
STPP (0.32%)	2.13
Sodium nitrite (90 ppm)	0.06

Make the volume of each treatment to 100 ml with water. STTP=Sodium tripolyphosphate, RBMLs=Restructured buffalo meat loaves

Four batters were prepared by replacing the lean buffalo meat with calcium lactate powder at 0, 1%, 1.25%, and 1.5% level. Once each mixing time was achieved, the meat batter was unloaded from the mixer, weighed and stuffed into stainless steel molds. Molds were squeezed with the wooden press to remove air pockets, closed tightly and placed in a pressure cooker filled with 1/3 boiling hot water and then cooked by steam without pressure. Slow heating rate was ensured by adjusting the flame regulating knob (Code: 637470, Regalia, Sun flame) to low so that the required internal temperature of 85°C of the product was achieved. The cooked meat block is cooled to room temperature, sliced into fillets, packaged into LDPE bags and analyzed for different parameters including sensory evaluation. The formulation of pre-standardized control RBMLs is shown in Table-3.

**Table-3:** Formulation of pre-standardized control RBMLs.

Ingredients	Amount required for 100 g
Lean meat (g)	77.0
Curing solution (ml)	15.0
Refined wheat flour (g)	3
Spices (g)	2.0
Condiments (g)	3.0
Total	100

### Analytical procedures

The pH of the cooked RBML was determined as per Trout *et al*. [28] method. 10 g of sample was homogenized with the help of ultra turrax tissue homogenizer (T-25 Germany) for about a minute in 50 ml of distilled water. The pH was recorded by immersing the electrode of a pH meter (model CP 901, Century Instrument Ltd., India) directly into the meat suspension. Cooking yield was determined by dividing cooked product weight by the raw uncooked weight and multiplying it by 100 to express as percent. The moisture, protein, fat, and ash content of the product were determined by standard methods using hot air oven, Kjeldahl assembly, Soxhlet extraction apparatus, and Muffle furnace, respectively, as per AOAC [29].

The calorific value of the sample was calculated using Gallenkamp and ballistic bomb calorimeter [30]. Approximately 1-2 pieces of meat sample were taken and weighed along with pre-weighed steel crucible. This crucible was placed on the support pillar in the base of the bomb. The firing wire and the sample were connected with the help of cotton thread. The bomb was fired under an oxygen pressure of 25 atm. The initial and final temperature readings on the galvanometer were noted. The deflection on the galvanometer was compared with 1 g standard benzoic acid of known calorific value (6.318 Kcal/g). The calorific value of the sample was calculated and expressed as Kcal/g.

Shear force value was determined as per the method described by Berry and Stiffler [31]. It is measured as the force required for shearing 1 cm^2^ block on Warner-Bratzler Shear Press (81031307 GR Elec. MFG. Co., USA) and expressed in kg/cm^2^. The calcium in the fortified RBMLs was estimated as per Talpatra *et al*. [32]. Water activity of functional RBMLs was measured by Aqua LAB dew point water activity meter 4TE (Decagon Devices Inc., United States). The samples were run in triplicate and the water activity meter was calibrated at regular intervals.

### Lovibond^®^ tintometer color units

The color of RBMLs was measured using a Lovibond^®^ tintometer (Model F, Greenwich, UK). Samples were cut with the help of scissors to the inner diameter of the sample holder and secured against the viewing aperture. The sample color was matched by adjusting the red (a*) and yellow (b*) values while keeping the blue unit fixed at 0.1. The corresponding color units were recorded. The hue angle and chroma values were determined using the formulae, tan^−1^(b/a) [33] and (a^2^+b^2^)^1/2^ [34], respectively, where a = red unit, b = yellow unit.

### Texture profile analysis (TPA)

The texture profile of RBMLs was measured with the help of instrumental TPA (TA.HDplus Texture Analyzer, Stable Micro Systems Ltd., UK). The procedure used for instrumental TPA was similar to those described by Bourne [35]. Chilled samples were tempered to bring to room temperature and then cut into 1 cm^2^. The samples were placed on a platform in a fixture and compressed twice to 85% of their original height by a compression probe (P75) at a crosshead speed of 10 mm/s through a two cycle sequence, using a 50 kg load cell. The calculation of TPA values was obtained by graphing a curve using force and time.

### Sensory evaluation

Seven semi-trained experienced taste panel consisting of scientists and post-graduate students of the Division of LPT, IVRI, Izatnagar, India were involved in conducting the sensory evaluation of the product. Panelists were trained following the procedure of Means and Schmidt [36]. The sensory panel was organized around 3.30-4.00 PM every time. Loaves were cooked as described and served to panelists immediately after cooking. In every session, the products were evaluated for general appearance, saltiness, flavor, texture, binding, juiciness and overall acceptability using 8-point descriptive scale [37], where 8 is extremely desirable, and 1 is extremely undesirable. Each panelist was supplied with a plate, a knife, a fork, a glass of cold tap water, and a disposal cup. Panelists received = 30 g of products from each treatment. They were asked to rinse their mouths with cold tap water before evaluating each sample.

### Statistical analysis

Three trials were conducted for each experiment in duplicate. The data generated from various trials under each experiment were pooled and analyzed by statistical method of one-way - analysis of variance and mean ± standard error using SPSS Statistics (version 20.0) software package developed as per the procedure of Snedecor and Cochran [38] and means were compared by using Dunkan's multiple range test [39].

## Results and Discussion

### Physico-chemical properties

The pH of the RBML with different levels of calcium lactate was found to be significantly lower (p<0.01) than that of control. There was a significant decline (p<0.01) in pH of the FRBML with each subsequent increase in the level of calcium lactate (Table-4). It may be attributed to acidic nature of the calcium lactate. The pH-reducing effect of calcium lactate in meat products was also documented by Alahakoon *et al*. [40], Devatkal and Mendiratta [19], and Caceres *et al*. [2].

**Table-4:** Effect of calcium lactate incorporation on the physico-chemical properties of calcium fortified RBMLs.

Parameters	Control	Level of calcium lactate incorporation		

		1%	1.25%	1.5%
Cooking yield (%)	93.07±0.30^a^	92.53±0.29^a^	91.96±0.33^a^	87.47±0.54^b^
Product pH	6.22±0.01^a^	6.10±0.01^b^	6.07±0.01^c^	5.90±0.02^d^
Moisture (%)	68.75±0.08^a^	68.23±0.11^b^	67.79±0.15^c^	65.99±0.46^d^
Protein (%)	19.57±0.10^a^	18.26±0.05^c^	18.08±0.09^c^	18.54±0.08^b^
Moisture protein ratio	3.19±0.02^c^	3.51±0.02^b^	3.73±0.01^a^	3.75±0.01^a^
Fat (%)	3.09±0.04^a^	2.77±0.03^bc^	2.74±0.03^c^	2.84±0.02^b^
Ash (%)	2.93±0.05^d^	3.11±0.03^c^	3.27±0.03^b^	3.42±0.02^a^
Calorific value (Kcal/100 g)	132.61±0.80^a^	130.97±0.52^b^	129.46±0.75^b^	127.99±0.82^b^
Shear force value (Kg/cm^2^)	0.75±0.05^a^	0.66±0.05^ab^	0.68±0.05^ab^	0.57±0.05^b^
Calcium concentration (mg/100 g)	6.48±0.32^d^	135.02±2.35^c^	165.73±4.29^b^	203.85±2.95^a^

Mean±SE with different superscripts in a row differ significantly (p<0.05), n_1_ (cooking yield and product pH)=3, n_2_ (proximate analysis)=6, n_3_ (shear force value)=30 for each treatment. SE=Standard error, RBMLs=Restructured buffalo meat loaves

Cooking yield of RBML fortified with calcium lactate was significantly low (p<0.01) at 1.5% treatment level as compared to the control. However, the values at 1% and 1.25% level of calcium lactate in treatment product remained comparable among themselves and control, with a gradual decline (Table-4). Similar kind of decrease in pH and cooking yield was also reported by Sofos [41] in the meat product. In general, a decrease in moisture content was observed with the increase in the level of calcium lactate. The moisture percentage of control product was significantly higher (p<0.01) than treatment groups. Decrease in moisture percentage could be due to the property of calcium to compete with phosphates for protein binding sites and thus limiting protein-phosphate water interaction resulting in more water loss and less moisture in product [20]. However, Devatkal and Mendiratta [19] reported a significant improvement (p<0.05) in moisture content of restructured pork rolls due to the incorporation of different levels (0.4%, 0.7%, and 1.0%) of sodium alginate with 0.3% calcium lactate.

There was a significant increase (p<0.05) in the crude protein percentage with increase in the level of calcium lactate from 1% to 1.5% level. This might be due to decrease in the cooking yield of the product with increase of calcium lactate level. Some decrease (p<0.05) in protein percentage in the fortified products as compared to control may also be attributed to replacement of lean in the formulation (Table-4). Similar findings have been reported by Naveena *et al*. [42] in microwave cooked chicken patties incorporated with calcium lactate and in cooked meat sausage enriched with calcium lactate by Caceras *et al*. [2]. The significantly higher (p<0.05) moisture protein ratio for RBML prepared with calcium lactate as compared to control is self-explanatory, the value being dependent on the moisture and protein percentages of the product. The fat percentage of the RBML fortified with calcium lactate was significantly lower (p<0.01) than control. This could also be due to the replacement of lean meat and presence of calcium lactate in the formulation. Caceras *et al*. [2] also reported a decrease in fat content in meat sausage with an increase in the level of calcium lactate. The values of ash content in treatment products increased significantly (p<0.01) with increasing level of calcium lactate. This increase could probably be due to direct addition of calcium salt. Increase in ash content in microwave cooked chicken patties and cooked sausages incorporated with calcium lactate were reported by Naveena *et al*. [42] and Caceras *et al*. [2], respectively.

The shear force values of the RBML fortified with calcium lactate were comparable (p>0.05) to the control up to 1.25% level. There was a significant decrease (p<0.05) in shear force value at 1.5% level in RBML as compared to control and other treatments (Table-4). Fortification with calcium lactate might interfere with protein-protein interactions or protein solubility, which could reduce shear force value. Similar findings have also been reported by Daengprok *et al*. [15] in Nhams (Thai-style fermented pork sausage). The calcium content in the fortified RBML was found to be significantly higher (p<0.01) than control. There was a significant increase (p<0.01) in the calcium concentration of treatment product with increase in calcium lactate level. Similar increase in the concentration of calcium had been reported by Daengpork *et al*. [15] and Caceres *et al*. [2] in Nhams and meat sausage fortified with calcium lactate, respectively. Water activity of the fortified RBML was found to be significantly lower (p<0.05) than control product, although both fortified and control product had high water activity due to high moisture content.

### Lovibond^®^ tintometer color units

The value of a* which denotes Lovibond^®^ tintometer color score for redness in fortified RBML was significantly higher (p<0.01) than the control product. The increase in redness value in calcium lactate fortified products might be due to a reduction in myoglobin denaturation. Devatkal and Mendiratta [19] found that addition of calcium lactate along with phosphate decreased the metmyoglobin accumulation in restructured pork rolls (Table-5).

**Table-5:** Lovibond^®^ tintometer color units and water activity of RBMLs.

Parameters	Control	Level of calcium lactate incorporation		

		1%	1.25%	1.5%
Redness (a*) value	8.28±0.05^d^	9.57±0.13^c^	10.97±0.24^b^	11.68±0.09^a^
Yellowness (b*) value	6.28±0.05^c^	7.42±0.11^b^	8.98±0.04^a^	9.20±0.11^a^
Hue angle	37.18±0.35^b^	37.79±0.64^ab^	39.36±0.70^a^	38.21±0.19^ab^
Chroma	10.40±0.30^d^	12.11±0.10^c^	14.18±0.16^b^	14.87±0.14^a^
Water activity (a_w_)	0.986±0.00^a^	0.976±0.00^b^	0.974±0.00^c^	0.971±0.00^d^

Mean±SE with different superscripts in a row differ significantly (p<0.05), n=6 for each treatment. SE=Standard error, RBMLs=Restructured buffalo meat loaves

The value of Lovibond^®^ tinometer color unit for yellowness denoted by b* was found to be significantly higher (p<0.01) in the RBML fortified with calcium as compared to control product. Although, the calcium lactate did not play a very important role in color development but a minor role of calcium lactate in enhancing the color stability of muscle could appear to be through the influx of lactate into the system. A similar result has also been reported by Devatkal and Mendiratta [19] in restructured pork rolls incorporated with calcium lactate. They found that enhancement with calcium resulted in less metmyoglobin discoloration and higher a* and b* values. Naveena *et al*. [42] also reported an increase in both a* and b* value in cooked chicken patties containing lactate as compared to control. Daengprok *et al*. [15] reported a non-significant increase (p>0.05) in yellowness (b*) value with an increase in the level of commercial grade calcium lactate in calcium fortified *Nhams*.

Hue angle and chroma values were derived values and thus obtained according to their corresponding redness and yellowness values. The hue angle of the fortified RBML was found to be significantly higher than control product. Contrary findings were reported by Caceres *et al*. [2] with an increase in the hue value in control as compared to calcium lactate-treated cooked meat sausages. Naveena *et al*. [42] also reported a lower value for hue in the cooked chicken patties containing calcium lactate than control.

Chroma, which indicates the intensity of the color, was found to be significantly higher (p<0.01) in the calcium fortified RBML than the control product. The increase in intensity could be due to the color stabilization by calcium lactate in the fortified product. Similar results were also observed by Naveena *et al*. [42] in cooked chicken patties containing calcium lactate.

### TPA

The hardness value for calcium fortified RBML was found to be significantly higher (p<0.01) as compared to control. The increase in hardness value with increased calcium lactate level could be due to forming bonds between meat proteins, in the presence of calcium, mainly myosin and favoring the formation of a stronger network that led to higher firmness [4]. An increase in hardness value with the incorporation of calcium lactate in both conventional and reduced fat cooked meat sausages was also reported by Caceres *et al*. [2]. Further, the hardness value could be higher (p<0.01) in the fortified RBML than control because of the lower moisture content in the fortified product. Several workers had reported a decrease in product hardness with an increase in moisture content [43-45].

There was a significant increase (p<0.01) in adhesiveness from control to fortified RBML with an increase in the level of calcium lactate, which might be attributed to better gelling by the salts of calcium present in the fortified product. Similar results were also reported by Caceres *et al*. [2] in reduced fat cooked meat sausages but in the case of conventional fat cooked meat sausages, they reported a decrease in the value of adhesiveness. Springiness, cohesiveness, and gumminess values did not show significant differences (p>0.05) between control and the fortified RBML. Verma *et al*. [46] also observed no significant (p>0.05) effect on cohesiveness in low-fat chicken nuggets due to the variation of contents. However, Ambadkar [47] and Caceres *et al*. [2] also reported an increase in values of both chewiness and gumminess in meat sausage and cooked buffalo meat salami respectively due to the incorporation of calcium lactate. Devatkal and Mendiratta [19] also reported an improvement in the texture profile of restructured pork rolls due to the addition of calcium lactate. Mehta [45] also found a similar result in fortified restructured chicken patties (Table-6).

**Table-6:** Instrumental TPA of RBMLs.

Parameters	Control	Level of calcium lactate incorporation		

		1%	1.25%	1.5%
Hardness (N/cm^2^)	46.62±1.07^c^	51.28±0.53^b^	55.04±1.30^a^	56.06±1.51^a^
Adhesiveness (Ns)	−0.17±0.00^c^	−0.16±0.00^bc^	−0.15±0.01^b^	−0.13±0.01^a^
Springiness (cm)	0.45±0.01	0.44±0.01	0.44±0.02	0.44±0.04
Cohesiveness (ratio)	0.31±0.01	0.26±0.05	0.31±0.01	0.281±0.01
Gumminess (N/cm^2^)	14.47±0.41	13.09±2.24	16.88±0.55	15.57±0.85
Chewiness (N/cm)	6.41±0.14	5.75±1.05	7.37±0.53	6.57±0.77

Mean±SE with different superscripts in a row differ significantly (p<0.05), n=6 for each treatment. SE=Standard error, RBMLs=Restructured buffalo meat loaves, TPA=Texture profile analysis

### Sensory evaluation

Sensory scores for the general appearance of the RBML incorporated with 1.0% and 1.25% calcium lactate were compared to the control, in spite of marginally lower (p>0.05) scores. There was a significant reduction (p<0.05) in general appearance score of RBML fortified with 1.5% calcium lactate. Mehta [45] also reported the similar trend, in general, appearance scores of low-fat chicken meat patties. The flavor scores for the RBML gradually decreased with increased level of incorporation and were significantly low (p<0.05) at 1.25% and 1.5% (incorporation levels) as compared to control. The flavor scores of RBML were comparable (p>0.05) at 1% and 1.25% incorporation levels. The gradual decrease in flavor scores of RBML with an increase of calcium lactate incorporation could be due to poor solubility of calcium lactate, imparting its own flavor. Brewer *et al*. [48] reported an intense flavor in fresh pork sausage containing 2.0% and 3.0% lactate than in sausage containing 0% or 1.0% of the lactate. Got *et al*. [49] also reported that 0.3 M level of calcium lactate brought about flavor and taste defects. Contrary to this, Daengprok *et al*. [15] found that fortifying Nhams (Thai-style fermented pork sausage) with either commercial or egg shell calcium lactate did not change the perception of flavor compared to control (Table-7).

**Table-7:** Effect of calcium lactate incorporation on the sensory attributes of RBMLs.

Sensory attributes	Control	Level of calcium lactate incorporation		

		1%	1.25%	1.5%
General appearance	7.14±0.05^a^	7.05±0.04^a^	7.03±0.03^a^	6.85±0.05^b^
Flavor	7.10±0.06^a^	6.97±0.07^ab^	6.90±0.05^b^	6.71±0.05^c^
Juiciness	7.16±0.05^a^	7.03±0.06^a^	7.03±0.05^a^	6.67±0.07^b^
Texture	7.18±0.05^a^	7.03±0.05^a^	7.03±0.05^a^	6.76±0.07^b^
Binding	7.19±0.06^a^	7.06±0.05^ab^	7.10±0.03^b^	6.78±0.06^c^
Saltiness	7.12±0.05	7.00±0.05	7.02±0.04	6.98±0.06
Overall acceptability	7.16±0.06^a^	7.02±0.05^a^	7.01±0.05^a^	6.72±0.07^b^

Mean±SE with different superscripts in a row differ significantly (p<0.05), n=21 for each treatment. SE=Standard error, RBMLs=Restructured buffalo meat loaves

There was a gradual decrease (p>0.05) in juiciness score with increased level of calcium lactate but the scores remained comparable to control up to 1.25% incorporation. RBML prepared with 1.5% calcium lactate level had significantly lower (p<0.05) juiciness score as compared to control. This might be attributed to decrease in the moisture content in the RBML. This finding was in accordance with Ambadkar [47] who also reported a decrease in juiciness perception with an increase in the level of calcium lactate. However, Caceras *et al*. [2] reported an increase in juiciness perception for the meat sausage enriched with calcium using calcium lactate as a source of calcium. A decrease in juiciness score with increased the level of supplementation of calcium was also reported by Boyle *et al*. [14] and Mehta [45].

The texture scores for the RBML followed almost the same trend as that of flavor. The texture score of the product incorporated with 1.5% calcium lactate was significantly lower (p<0.05) than others, whereas up to 1.25% incorporation, the scores were comparable to control as well as 1.0% incorporation. A significant decrease (p<0.05) in texture score of low-fat meat sausage enriched with calcium was also reported by Caceras *et al*. [2]. Daengprok *et al*. [15] also reported that calcium fortified Nhams were less firm than control. Binding scores also showed a significant reduction (p<0.05) with increased incorporation level of calcium lactate. The sensory score for the saltiness of control was slightly higher (p>0.05) as compared to the fortified RBML. This was due to the taste of calcium lactate in the treatment products. The scores for the treatment products were marginally (p>0.05) different among themselves. The slightly sour taste of calcium lactate was masked by the addition of sucrose in the curing solution.

**Figure-2:**
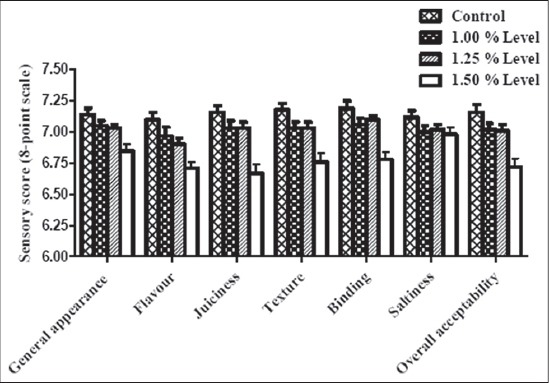
Effect of calcium lactate incorporation on the sensory attributes of restructured buffalo meat loaves.

Overall acceptability scores of RBML declined gradually with increase in the incorporation level of calcium lactate. RBML with 1.5% calcium lactate had significantly lower (p<0.05) overall acceptability score as compared to control and fortified RBML with 1.25% calcium lactate incorporation, which remained comparable. The overall acceptability pattern reflected the related sensory rating for flavor, texture, juiciness, and saltiness of the products. Caceres *et al*. [2] also reported a lower overall acceptability score in cooked meat sausages enriched with calcium using calcium lactate as a source. Further, Mehta [45] also reported that the overall acceptability scores of fortified chicken patties gradually decreased with increase in incorporation level of calcium lactate (Figure-2).

## Conclusion

Present study concluded that fortification of calcium in RBML is vital since buffalo meat is deficient in calcium. The addition of calcium lactate up to 1.25% (i.e., 15% recommended daily allowance), in RBML, improve the product color and textural properties besides maintaining sensory and physico-chemical attributes of the product.

## Authors’ Contributions

IA and BDS planned the study. IA carried out the study with assistance from BDS and SRA. ST and OPM revised the draft manuscript prepared by IA and AK. All authors read and approved the final manuscript.
